# Death from Erysipelas

**Published:** 1842-05

**Authors:** James Mease

**Affiliations:** Philadelphia


*Death from Erysipelas: Pricks by a Scalpel—Slight Razor Cut—Scratch by a Nail—Pulling out a Mole—Remedy for Bee Stings.* By James Mease, M. D., of Philadelphia.—In the month of December last, it was stated in the newspapers, that a young man had died at Framingham, Connecticut, in consequence of using the same razor, (without wiping) to shave himself, which he had just before employed to shave his deceased father. The impression was, that some morbid matter on the face of the father had been applied to that of the son, and had caused his death. The principle of action—*humani nihil à me alienum puto,* might have been sufficient for me to inquire about the fact, but more was concerned than the indulgence of professional curiosity, or a benevolent feeling:—a life was in question; I therefore determined to ascertain the particulars, and accordingly addressed a letter to a gentleman resident in the town, requesting a communication of them. From him I received the following printed article, by the attending physician, Dr. Whitney, which appears to have been addressed to the editor of the newspaper, published in the place where the parties resided.

"*Framingham, Oct.* 29, 1841.

"Mr. Editor.—Having seen different accounts in the newspapers respecting the death of our very worthy and esteemed townsman, (Mr. Henry W. Coolidge) and knowing the circumstances of the case, I take the liberty of stating them to you as they occurred. The aged father, who had been sick for some time, died of a hepatic disease. He had too, as is frequently the case, at the termination of many diseases, a very bad thrush or canker in the mouth, attended with frequent hemorrhages. The son, who had always enjoyed good health and was well at this time, (with the exception of what he termed a cold sore or two on his lip) shaved the father after his decease. Then with the same razor, brush and soap, he shaved himself immediately. This was on Tuesday, and by the Saturday following, one of the sores upon the lip became very troublesome, affecting it internally and externally. On Sunday his disease progressed, and thus continued daily to increase, producing a most hideous swelling of the head and face, till the next Saturday, when he died. The disease was no doubt gangrenous erysipelas. Was it an idiopathic disease, or was it communicated from the father by means of the brush? Should think the latter highly probable.

"Yours, with respect, "S. W."

I presume the sores on the lip must have been irritated by the edge of the razor, and given rise to the erysipelas. That the cause suggested was quite sufficient to produce this often unmanageable disease, is probable from our knowledge of its frequent occurrence after apparently slight local affections, and of serious consequences, and even death from them. Many anatomists have died from the irritation roused in the system by pricking a finger with a scalpel. Falck, an English author on syphilis, and of other medical works, and two professors of the Medical School in Dublin, died from the effects of the first accident. Falck, many years since, and the two last within ten or twelve years. Another lost his life from scratching his hand with the sharp edge of a rib. A few weeks since, a man cut one of his fingers when strapping his razor, and died shortly after, from inflammation and gangrene in his hand and arm. Another scratched his finger last year, with a nail in a barrel which he was handling, and died in the same way. I knew a dreadful and mortal cancer which destroyed the lower jaw, and finally life, to follow the pulling out a small mole in the chin; and I have recorded several cases of death from the poison of honey bees, an humble bee, and wasps, which they had manufactured from the sugar, or saccharine fluid sucked by them from flowers, fruits, or sweet cider. Death took place in 10, 20, and 30 minutes after the stings were inflicted.* The poison of spiders has been equally mortal as that of bees. But what was the modus operandi of these venoms? why, you will say, they *destroyed the vital principle*,—certainly,—but what is this? the answer leaves the inquirer as wise as before he asked the question. Here is a fine and new subject for a prize essay, by some learned medical corporate body. By the way, since the publication of my paper just referred to, a case occurred in the vicinity of Philadelphia, of a bee-sting which was attended with spasms and such high excitement as to require venesection twice. Aqua ammonia was also liberally given with good effect. This remedy should be applied immediately to the part after a sting, and also given internally, every ten or fifteen minutes, in doses of 10 or 15 drops in water, and the vapor inhaled by the nostrils.—*Am. Med. Intelligencer.*

* Amer. Journal of Med. Sciences, for Nov. 1836.

